# Neutralization of interleukin-38 exacerbates coxsackievirus B3-induced acute myocarditis in mice

**DOI:** 10.1186/s12985-021-01687-w

**Published:** 2021-11-14

**Authors:** Yimin Xue, Mingguang Chen, Qian Chen, Tingfeng Huang, Qiaolian Fan, Fenghui Lin, Jun Ke, Feng Chen

**Affiliations:** 1 Shengli Clinical Medical College of Fujian Medical University, Fuzhou, 350001 Fujian People’s Republic of China; 2 The Fourth Department of Intensive Care Unit, Fujian Provincial Hospital, Fuzhou, 350001 Fujian People’s Republic of China; 3 Department of Emergency, Fujian Provincial Hospital, Fuzhou, 350001 Fujian People’s Republic of China; 4 Fujian Provincial Key Laboratory of Emergency Medicine, Fuzhou, 350001 Fujian People’s Republic of China

**Keywords:** Acute viral myocarditis, IL-38, Th1 cells, Th17 cells, Coxsackievirus B3

## Abstract

**Background:**

Interleukin (IL)-38, a novel member of the IL-1 family, has been reported to be involved in several diseases associated with viral infection. However, the expression and functional role of IL-38 in acute viral myocarditis (AVMC) have not been investigated.

**Methods:**

Male BALB/c mice were treated with intraperitoneal (i.p.) injection of coxsackievirus B3 (CVB3) for establishing AVMC models. On day 7 post-injection, the expression of IL-38 and IL-36R (IL-36 receptor) were measured. Mice were then treated with i.p. injection of mouse Anti-IL-38 Antibodies (Abs) for neutralization of IL-38. The survival, bodyweight loss, cardiac function, and myocarditis severity of mice were recorded. The percentages of splenic Th1 and Th17 cells, the expression levels of Th1/Th17-related master transcription factors (T-bet and RORγt) and cytokines were determined by flow cytometry, RT-qPCR, and ELISA, respectively. Cardiac viral replication was further detected.

**Results:**

The mRNA and protein expression levels of IL-38 in myocardium and serum, as well as cardiac IL-36R mRNA levels were significantly elevated in mice with AVMC. Increased IL-38 levels were negatively correlated with the severity of AVMC. Neutralization of IL-38 exacerbated CVB3-induced AVMC, as verified by the lower survival rate, impaired cardiac function, continuous bodyweight loss, and higher values of HW/BW and cardiac pathological scores. In addition, neutralization of IL-38 suppressed Th1 cells differentiation while promoted Th17 cells differentiation, accompanied by decreased T-bet mRNA expression and increased RORγt expression. Down-regulation of IFN-γ and up-regulation of IL-17, TNF-α, and IL-6 mRNA and protein expression levels in myocardium and serum were also observed in the IL-38 neutralization group. Furthermore, neutralization of IL-38 markedly promoted cardiac viral replication.

**Conclusions:**

Neutralization of IL-38 exacerbates CVB3-induced AVMC in mice, which may be attributable to the imbalance of Th1/Th17 cells and increased CVB3 replication. Thus, IL-38 can be considered as a potential therapeutic target for AVMC.

**Supplementary Information:**

The online version contains supplementary material available at 10.1186/s12985-021-01687-w.

## Background

Acute viral myocarditis (AVMC), a common inflammatory myocardial disease, is one of the leading causes of acute-onset heart failure and sudden death among young patients [1, 2]. Some patients with AVMC may progress into chronic myocarditis, causing continuous destruction of cardiomyocytes and the initiation of remodeling process [3]. Coxsackievirus B3 (CVB3), a member of the Picornaviridae family, has been identified as the most common pathogen for AVMC [4]. Although a multitude of studies have demonstrated that the major pathogenesis of AVMC is excessively uncontrolled inflammatory responses triggered by myocardial damage following viral infection, the direct attack of the virus on cardiomyocytes is also considered a critical component for AVMC development and progression [5, 6]. Accumulating evidence has revealed that both T helper (Th) 1 and Th17 cells play a crucial role in the early immune reaction to CVB3 infection [7–9]. However, up to now, the treatment of AVMC is still mostly restricted to supportive measures as there is no specific treatment for the disease. It is essential to clarify the fundamental mechanisms responsible for AVMC.

Interleukin (IL)-38 (IL-1F10), a newly identified cytokine of the IL-1 family, is located in the IL-1 family cluster on chromosome 2 next to the genes encoding IL-1 receptor antagonist (IL-1Ra) and IL-36Ra, and shares nearly 41% homology with IL-1Ra and 43% homology with IL-36Ra [10]. Similar to other IL-1 family members, IL-38 lacks a signal peptide and has no caspase-1 consensus cleavage site [10, 11]. It mediates its biological effects via the specific receptor IL-36R, a partial receptor antagonist of IL-36. Therefore, IL-38 plays an antagonistic role in the pro-inflammatory effects of IL-36 cytokines, including IL-36α, IL-36β, and IL-36γ [12]. IL-38 is an abundant, ubiquitously expressed cytokine, which can be secreted by fibroblast-like synoviocytes, peripheral blood mononuclear cells (PBMCs), keratinocytes, epithelial cells, and various immune cells, such as B cells, natural killer (NK) cells, macrophages, and dendritic cells (DCs) [11, 12]. Abnormal expression of IL-38 has been found in many inflammatory diseases, including rheumatoid arthritis (RA), allergic asthma (AA), and systemic lupus erythematosus (SLE) [13–15]. In addition, elevated serum IL-38 levels at baseline can be used to predict virological response in telbivudine-treated patients with chronic hepatitis B [16]. Moreover, Gao and Conti have reported that IL-38 is a potential therapeutic cytokine that inhibits inflammation in viral infections, including those caused by the influenza virus and Coronavirus-19 [17, 18]. These findings indicate that IL-38 may play an important role in the pathogenesis of diseases associated with viral infection. However, the expression and functional role of IL-38 in AVMC are still largely unknown.

To tentatively tap this field, we investigated the expression of IL-38 and IL-36R in vivo in mice with AVMC. The percentages of splenic Th1 and Th17 cells, the expression levels of Th1/Th17-related master transcription factors (T-bet and RORγt) and cytokines, and cardiac viral replication were determined. Additionally, neutralizing Anti-IL-38 Antibodies (Abs) were given to AVMC mice to further explore the effect of IL-38 on myocarditis.

## Methods

### Mice

Specific pathogen-free grade wild-type male BALB/c mice aged 6 weeks (production license: SCXK (Zhe) 2019-0001) were purchased from the Zhejiang Vital River Laboratory Animal Technology Co., Ltd. (Zhejiang, China). Mice were housed under standard pathogen-free conditions at the Experimental Animal Center (Fujian Academy of Medical Sciences, Fuzhou, Fujian, China). All studies were approved by the Animal Experimentation Ethics Committee of Shengli Clinical Medical College of Fujian Medical University (SYXK: 2016-0004). The experimental methods were performed in accordance with the approved guidelines.

### CVB3 infection

The CVB3 (Nancy strain, a kind gift from Prof. Weifeng Wu of Guangxi Medical University) was produced in monolayers of HeLa cells (ATCC, CCL-2) and stored at −80 °C. The virus titer, determined based on a standard plaque-forming unit (PFU) assay on HeLa cells, was 1 × 10^8^ PFU/ml. Male BALB/c mice aged 6 weeks were infected by intraperitoneal (i.p.) injection of 200 μl phosphate-buffered saline (PBS) containing 2 × 10^5^ PFU of CVB3 to establish the AVMC models.

### Induction of AVMC

A total number of 28 BALB/c mice were randomly assigned into two groups as follows: (1) Control group (n = 10) containing mice treated with i.p. injection of PBS (200 μl per mouse); (2) AVMC group (n = 18) comprising mice administered CVB3. The day of i.p. injection was defined as day 0. All surviving mice were sacrificed on day 7 after CVB3 infection. The heart, spleen, and blood were collected.

### Neutralization of IL-38

To elucidate the role of IL-38 in vivo, Anti-IL-38 Abs were used to neutralize IL-38 in mice. We performed a dose–response experiment of Anti-IL-38 Abs in vivo based on the dose previously reported in the studies [19, 20]. As shown in Additional file 1: Figure S1, mice were treated with three different doses of Anti-IL-38 Abs (25, 50, or 75 μg per mouse) by i.p. injection on day 0 and day 4 after CVB3 infection. It was observed that Abs dose of 50 or 75 μg per mouse per injection was more effective in neutralizing IL-38 expression in vivo than that of 25 μg per mouse per injection (both *P* < 0.01), while there was no significant difference in effectiveness between the doses of 50 and 75 μg per mouse per injection (both *P* > 0.05). Since the Abs dose of 50 μg per mouse per injection was sufficient to effectively neutralize IL-38 expression in vivo, this dose was used for subsequent experiments. A total of 55 BALB/c mice were randomly divided into four groups: 1) IL-38 neutralization (IL-38N) group [mice treated with i.p. injection of Anti-IL-38 Abs (50 μg per mouse, on day 0 and day 4, n = 15) after CVB3 infection]; 2) IgG group [mice treated with i.p. injection of IgG_2A_ isotype control (50 μg per mouse, on day 0 and day 4, n = 15) after CVB3 infection]; 3) PBS group [mice treated with i.p. injection of PBS (50 μg per mouse, on day 0 and day 4, n = 15) after CVB3 infection]; and 4) Sham group [untreated mice administered an i.p. injection of PBS on day 0 and day 4 (n = 10)]. Mouse Anti-IL-38 Abs and IgG_2A_ isotype control were obtained from R&D Systems, Minneapolis, MN, USA. The survival rates and bodyweight changes were monitored daily until day 7. The spleen and heart were removed aseptically to be measured on day 7 post CVB3 infection. Before mice were killed, the cardiac function was assessed and then serum was collected for the study.

### Transthoracic echocardiography

The cardiac function of mice was evaluated non-invasively on day 7 by transthoracic echocardiography using a Vevo2100 Ultrasound System (Fujifilm VisualSonics, Bothell, WA, USA) equipped with a 40 MHz transducer-phased-array transducer. Mice were anesthetized with i.p. administration of sodium pentobarbital (1%, 50 mg/kg) and immobilized in a supine position. The parameters of heart function were recorded in two-dimensional mode, and left ventricular parasternal long-axis views were obtained in M-mode imaging at the papillary muscle level. Ventricular parameters, including left ventricular end-systolic diameter (LVESD), left ventricular end-diastolic diameter (LVEDD), fractional shortening (FS), and ejection fraction (EF) were measured and analyzed using a standard formula as described previously [21]. The sonographer was unaware of the group allocations.

### Histopathological examination

Heart ventricular tissues fixed in 10% phosphate-buffered formalin were embedded in paraffin, and sections (5 μm thick) were stained with hematoxylin and eosin (H&E) to quantify the severity of inflammation. Histopathological changes were viewed using a Leica DM2000 LED microscope (Leica, Wetzlar, Germany). Cardiac pathological scores were graded by two independent researchers in a blinded manner according to the following scoring method: 0, no inflammation; 1, 1–5 distinct mononuclear inflammatory foci with involvement of 5% or less of the cross-sectional area; 2, > 5 distinct mononuclear inflammatory foci, or involvement of over 5% but not over 20% of the cross-sectional area; 3, diffuse mononuclear inflammation involving over 20% of the area, without necrosis; and 4, diffuse inflammation with secondary necrosis and acute inflammation [22].

### Immunohistochemistry

For immunohistochemistry, the streptavidin–biotin complex method was applied. The staining was performed using a Streptavidin–Biotin Complex kit (Boster, Wuhan, Hubei, China) according to the manufacturer's instructions. The obtained sections of heart ventricular tissues were deparaffinized using xylene, followed by rehydration using alcohol and washing with PBS. The sections were microwaved in citrate buffer for 15 min to retrieve antigen and then treated with 3% hydrogen peroxide for 10 min to block endogenous peroxidase. The sections were treated with 10% goat serum (Boster) for 30 min at room temperature to block the binding of nonspecific Abs. Rabbit anti-mouse IL-38 polyclonal antibody (1: 200, Bioss, Beijing, China) was added to the sections, and incubation was performed overnight at 4 °C. Histochemical reactions were then developed using 3, 3-diaminobenzidine (Sigma-Aldrich, St. Louis, MO, USA) as the chromogenic substrate for peroxidase. Negative controls replaced the primary antibody with nonimmune goat serum, with all other steps performed as above. The semi-quantitative analysis of IL-38 deposition was performed using Image-Pro Plus 6.0 software (Media Cybernetics, Bethesda, MD, USA). For each slide, 5 randomly chosen fields at 400× magnification were photographed and analyzed by two independent pathologists uninformed of the experimental grouping to measure the integrated optical density (IOD).

### Flow cytometric analysis

The spleen from each mouse was removed aseptically and dispersed through nylon mesh to generate a single-cell suspension. After red blood cells removal by Red Blood Cell Lysing Buffer (Sigma-Aldrich), the splenic mononuclear cells were washed twice in RPMI 1640 (Invitrogen, Carlsbad, CA, USA) and resuspended in RPMI 1640 medium with 10% FBS (Invitrogen). Cells were stimulated with phorbol myristate acetate (25 ng/ml, Sigma-Aldrich) and ionomycin (1 μg/ml, Sigma-Aldrich) for 5 h at 37 °C in the presence of GolgiPlug (1 μl/10^6^ cells, BD Biosciences, Breda, the Netherlands). After stimulation, cells were collected, washed, and stained with phycoerythrin cyanine-5-conjugated anti-mouse CD4 (eBioscience, San Diego, CA, USA). Cells were stained intracellularly with anti-IFN-γ, or IL-17 mouse Abs conjugated with Alexa Fluor®488, or phycoerythrin after fixation and permeabilization according to the manufacturer's instructions (eBioscience), and then measured by flow cytometry using a FACSCalibur flow cytometer (BD Biosciences). The data were analyzed using the FCS Express 7 Research Edition software (DeNovo Software, Glendale, CA, USA).

### Quantitative real-time PCR (RT-qPCR)

Total RNA was extracted from cardiac tissue using TRIzol reagent (Invitrogen), followed by transcription into cDNA with a PrimeScript™ RT reagent kit (TaKaRa, Dalian, Liaoning, China) according to the instructions provided by the manufacturer. Primers for IL-38, IL-36R, IFN-γ, IL-17, TNF-α, IL-6, T-bet, RORγt, CVB3, and the housekeeping gene β-actin were designed with the Primer Premier 5.0 software (Premier Biosoft, Palo Alto, CA, USA) and are shown in Table 1. RT-qPCR was performed in an ABI 7500 Sequence Detection System (Applied BioSystems, Foster City, CA, USA) using a SYBR® Premix Ex Taq™ II kit (TaKaRa) for detection. The thermal cycling conditions were: initial denaturation at 94 °C for 3 min, followed by 40 cycles of 94 °C for 30 s, 60 °C for 30 s and 72 °C for 30 s. The relative expression of target genes was normalized to β-actin mRNA expression and quantified by the 2^−ΔΔCT^ method [23]. Reactions were performed in a 25 μl volume, and each sample was run at least in duplicate.

**Table 1:** Primers used in RT-qPCR

Primer name	Sequence (5′–3′)
IL-38	Forward: GTGAACATCGAGGACCTATACAAG
	Reverse: TCAGTATGGGTGGAGGGTTC
IL-36R	Forward: TGCTTCTGCTTTTCGTGGCAGCA
	Reverse: GCCCCGTTTGTTTCTGGCGG
IFN-γ	Forward: CTCAAGTGGCATAGATGTGGAAG
	Reverse: GCTGGACCTGTGGGTTGTTGA
IL-17	Forward: CTGTGTCTCTGATGCTGTT
	Reverse: TGGAACGGTTGAGGTAGT
TNF-α	Forward: AGTCCGGGCAGGTCTACTTT
	Reverse: TTGGACCCTGAGCCATAATC
IL-6	Forward: CCAGAAACCGCTATGAAGTTCC
	Reverse: TTGTCACCAGCATCAGTCCC
T-bet	Forward: AGCAAGGACGGCGAATGTT
	Reverse: GTGGACATATAAGCGGTTCCC
RORγt	Forward: TGCGACTGGAGGACCTTCTAC
	Reverse: TCACCTCCTCCCGTGAAAAG
CVB3	Forward: CGGTACCTTTGTGCGCCTGT
	Reverse: CAGGCCGCCAACGCAGCC
β-actin	Forward: AATTCCATCATGAAGTGTGA
	Reverse: ACTCCTGCTTGCTGATCCAC

### Plaque-forming assay

Plaque-forming assays and viral titers were performed using standard methods. A portion of heart tissue was weighed and then homogenized in 2 ml of sterile PBS, followed by three freeze/thaw cycles to lyse the cells and release the virus. After spinning at 450×*g* for 10 min, the supernatants were sequentially 10-diluted in RPMI 1640 medium. Virus-containing supernatants were overlaid on HeLa cell monolayers and incubated for 1 h at 5% CO_2_, 37 °C, in six-well plates. Cells were then washed 3 times with PBS and covered with 2 ml 0.4% agar containing RPMI 1640 and 10% FBS. To allow counting of the plaques, the cell monolayers were fixed and stained after 3 days of incubation. The viral titers were calculated as PFU per organ weight (in grams).

### Cytokine assay

The serum samples were obtained by centrifugation. The level of IL-38 in mouse serum was detected by using a Mouse IL-38 ELISA kit (SND-M144, Chuzhou Shinuoda Biological Technology Co., Ltd., Anhui, China), and the levels of IFN-γ, IL-17, TNF-α, and IL-6 in serum were detected by Mouse ELISA kits (ExCell Biology Inc., Shanghai, China) according to the instructions provided by the manufacturer. The lowest detectable concentrations of IL-38, IFN-γ, IL-17, TNF-α, and IL-6 were 7.5, 4, 7, 7, and 7 pg/ml, respectively. No significant cross-reactivity or interference was observed. All samples were measured in triplicate, and the mean value was calculated.

### Statistical analysis

Data are expressed as mean ± SD. Statistical analysis of data was performed by unpaired 2-tailed *t*-test or one-way ANOVA, as indicated. The difference in cardiac pathological scores was performed by the Mann–Whitney *U* test. Survival was estimated using the Kaplan–Meier method, and the difference in survival was compared by the log-rank test. Correlation between variables was determined by the Spearman rank test. All data were analyzed with GraphPad Prism 8 software (GraphPad Software, La Jolla, CA, USA). *P* < 0.05 was considered statistically significant.

## Results

### Assessment of AVMC severity

**Fig. 1:**
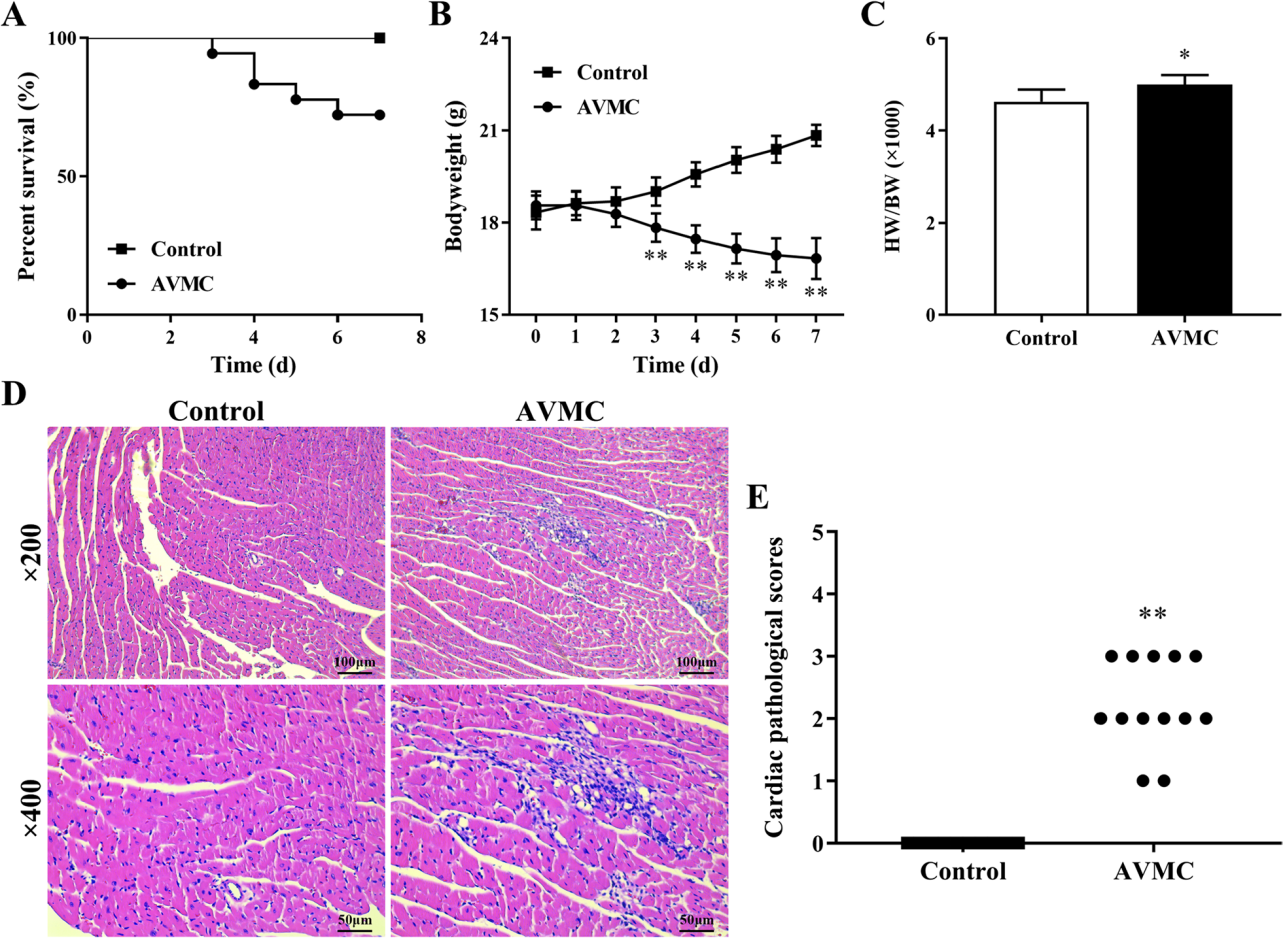
Assessment of AVMC severity. Male BALB/c mice were infected with CVB3 on day 0. The survival rates (**A**) and bodyweight changes (**B**) of mice in the control (n = 10) and AVMC (n = 18) groups were monitored daily until day 7. **C** Values of HW/BW in two groups were recorded on day 7. **D** Representative images of myocardial histopathology H&E staining in two groups (magnification × 200 and × 400). **E** Cardiac pathological scores in two groups. Each point represents an individual mouse. ^*^P < 0.05, ^**^P < 0.01, compared to control group

All mice in the control group grew normally without any apparent behavioral abnormalities. Starting on day 2, CVB3-treated mice showed the symptoms of AVMC, including weakness, coat ruffling, back arching, loss of appetite, fatigue, weight loss, and even death. The 7-day survival rates of mice after CVB3 infection were monitored. A total of 13 out of 18 (72.22%) mice survived in the AVMC group. In detail, a total of 1, 2, 1, and 1 mice died on day 3, 4, 5, 6 post-infection in the AVMC group, respectively. In contrast, none of the mice died in the control group (Fig. 1A). The bodyweights of AVMC mice significantly decreased compared to control mice from day 3 to day 7 (Fig. 1B, all *P* < 0.01). In addition, the values of heart weight/bodyweight (HW/BW) and cardiac pathological scores of heart sections in AVMC mice were dramatically higher than those of control mice on day 7 (Fig. 1C-E, P < 0.05 or *P* < 0.01).

### Elevated expression of IL-38 and IL-36R in AVMC

**Fig. 2:**
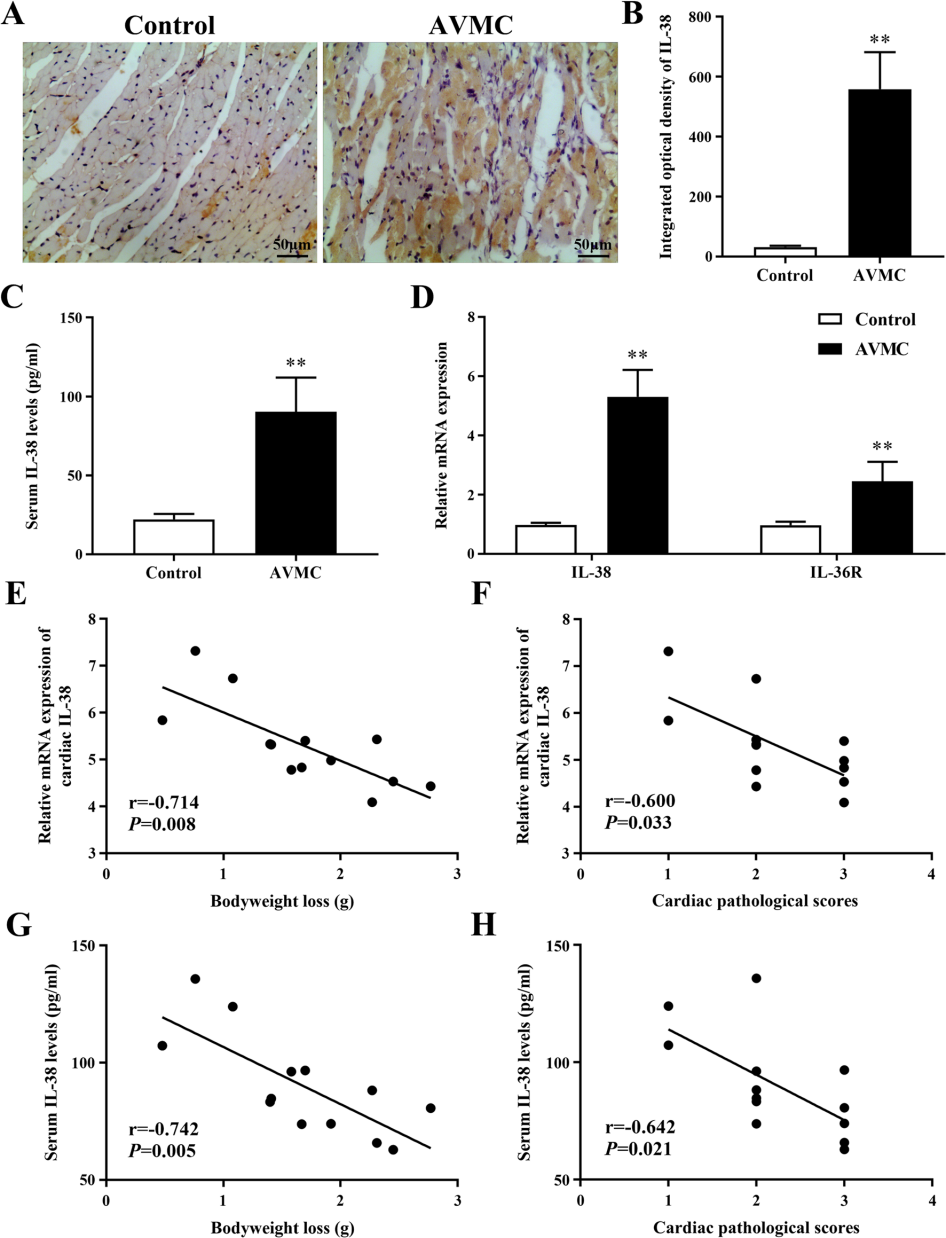
Elevated expression of IL-38 and IL-36R in AVMC. **A** Representative images of IL-38 immunohistochemistry of heart tissue from the control (n = 10) and AVMC (n = 13) groups (Brown granules, magnification × 400). **B** Morphometric quantitation of cardiac IL-38 protein expression. **C** ELISA analysis of serum IL-38 levels. **D** Relative cardiac mRNA expression of IL-38 and IL-36R detected by RT-qPCR. **E–F** Negative association of cardiac IL-38 mRNA expression with bodyweight loss and cardiac pathological scores on day 7 post-infection. Each point represents an individual mouse. **G–H** Negative association of serum IL-38 levels with bodyweight loss and cardiac pathological scores on day 7 post-infection. Each point represents an individual mouse. ^**^P < 0.01, compared to control group. Data are expressed as mean ± SD

On day 7 post-infection, the protein and mRNA expression levels of IL-38 were enhanced in the myocardium of AVMC mice, compared to those of control mice (Fig. 2A, B and D, both *P* < 0.01). As shown in Fig. 2C, the markedly increased level of circulating IL-38 protein was also observed in the AVMC group compared to that in the control group (*P* < 0.01). Furthermore, the cardiac relative mRNA expression of IL-36R, the specific receptor of IL-38, was significantly elevated in the AVMC group compared to that in the control group (Fig. 2D, *P* < 0.01).

### Association between IL-38 expression and the severity of AVMC

To explore the potential role of IL-38 in the pathogenesis of AVMC, we examined the association of IL-38 levels with disease severity, including bodyweight loss and cardiac pathological scores on day 7 post-infection. As shown in Fig. 2E–F, cardiac IL-38 mRNA expression was negatively correlated with bodyweight loss (r =  − 0.714, *P* = 0.008) and cardiac pathological scores (r =  − 0.600, *P* = 0.033). Meanwhile, similar results were obtained for the serum IL-38 levels (Fig. 2G–H), indicating that IL-38 may play a protective role in CVB3-induced AVMC.

### Neutralization of IL-38 reduced survival rate and impaired cardiac function

**Fig. 3:**
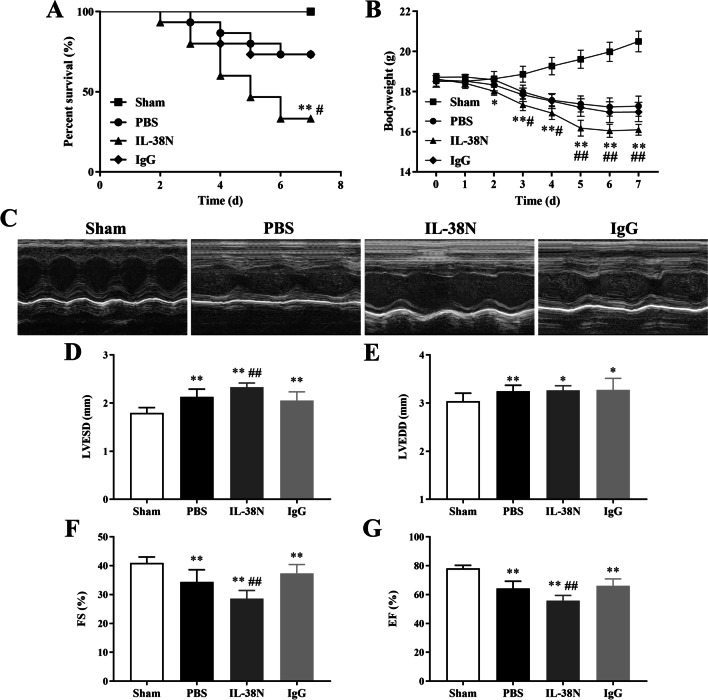
Neutralization of IL-38 reduced survival rate and impaired cardiac function. Male BALB/c mice were infected with CVB3 on day 0 and then treated with i.p. injection of PBS, Anti-IL-38 Abs, or IgG_2A_ isotype control on day 0 and 4 after CVB3 infection. **A** Survival analysis in the Sham (n = 10), PBS (n = 15), IL-38N (n = 15), and IgG (n = 15) groups after CVB3 infection.** B** The bodyweight changes were monitored daily until day 7. **C** Representative M-mode echocardiography images of the left ventricle in different groups on day 7 post-infection. **D–G** LVESD, LVEDD, FS, and EF were measured on day 7 post-infection. ^*^P < 0.05, ^**^P < 0.01, compared to Sham group; ^#^*P* < 0.05, ^##^*P* < 0.01, compared to IgG group. Data are expressed as mean ± SD

We further investigated the protective role of IL-38 against CVB3-induced AVMC by using Mouse Anti-IL-38 Abs in vivo. The number of mice surviving to day 7 was 10, 11, 5, and 11 for Sham, PBS, IL-38N, and IgG groups, respectively. The 7-day survival rates of the PBS and IgG groups both decreased to 73.33% (11/15), and a marked decline in survival rate (33.33%, 5/15) was observed in the IL-38N group compared to that of the IgG group (*P* = 0.037, Fig. 3A). Meanwhile, mice treated with Anti-IL-38 Abs suffered significant and continuous bodyweight loss from day 3 to day 7 (Fig. 3B, P < 0.05 or *P* < 0.01). To compare cardiac function in different groups, LVESD, LVEDD, FS, and EF were measured and analyzed by transthoracic echocardiography on day 7. As shown in Fig. 3C–G, compared to IgG_2A_ isotype control-treated mice, the echocardiographic assessment revealed that LVESD was larger, while FS and EF were lower in mice treated with Anti-IL-38 Abs (all *P* < 0.01). There was no statistical difference in LVEDD between the IL-38N and IgG groups (Fig. 3E, *P* > 0.05). Regarding the survival rates, bodyweight loss, LVESD, LVEDD, FS, and EF, no significant differences were observed between the PBS and IgG groups (all *P* > 0.05).

### Neutralization of IL-38 exacerbated CVB3-induced myocarditis

**Fig. 4:**
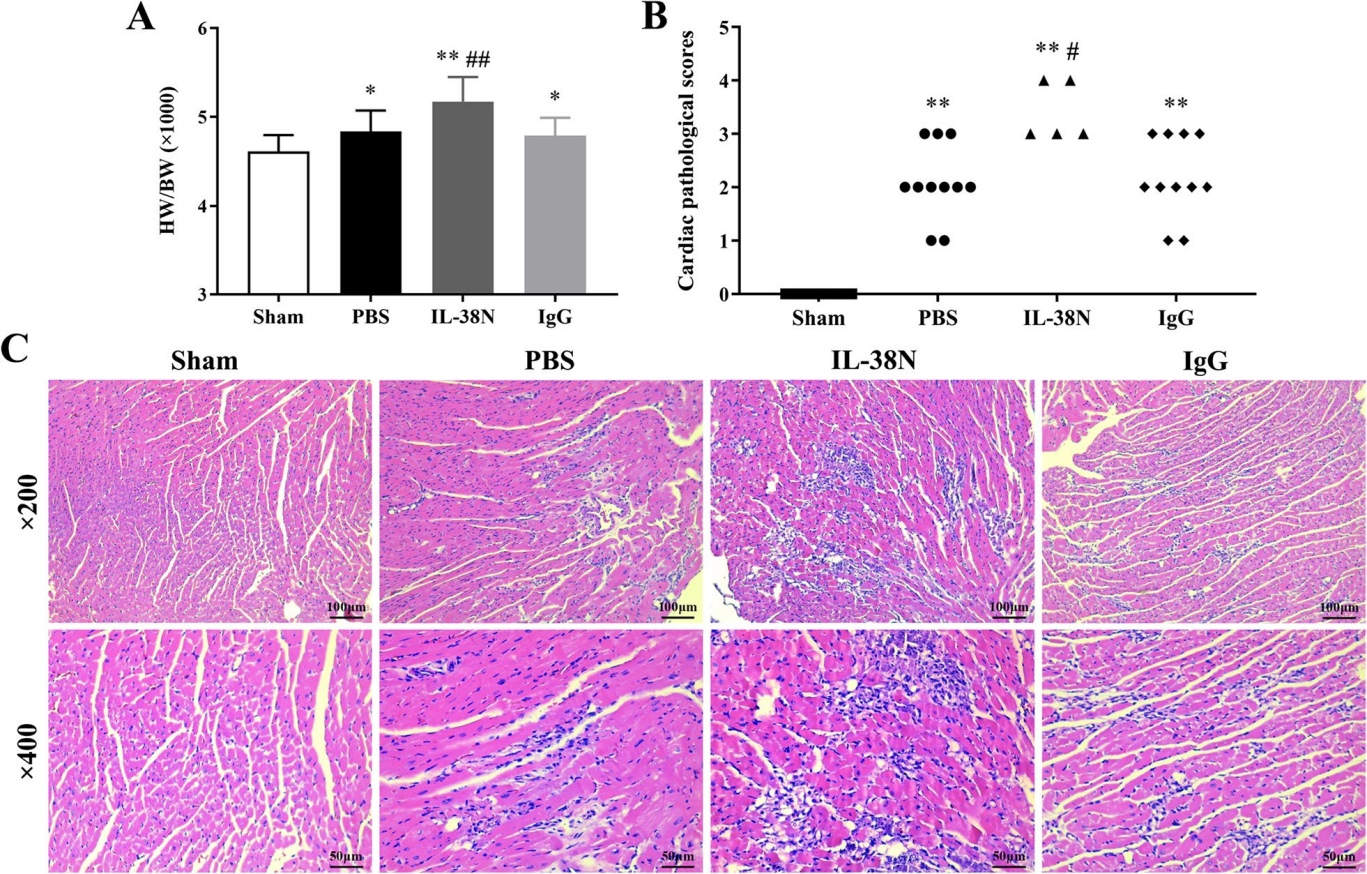
Neutralization of IL-38 exacerbated CVB3-induced myocarditis. **A** Values of HW/BW in the Sham (n = 10), PBS (n = 11), IL-38N (n = 5), and IgG (n = 11) groups. **B** Cardiac pathological scores in different groups. Each point represents an individual mouse. **C** Representative images of myocardial histopathology H&E staining in different groups (magnification × 200 and × 400). ^*^P < 0.05, ^**^P < 0.01, compared to Sham group; ^#^*P* < 0.05, ^##^*P* < 0.01, compared to IgG group

As shown in Fig. 4, mice treated with Anti-IL-38 Abs developed more severe myocarditis. Firstly, the values of HW/BW in mice treated with Anti-IL-38 Abs were higher than those in the IgG group on day 7 post-infection (Fig. 4A. *P* < 0.01). Additionally, the cardiac pathological scores of heart section in mice receiving Anti-IL-38 Abs were significantly increased compared to those in the IgG group (Fig. 4B–C, *P* < 0.05). There were no significant statistical differences between the PBS and IgG groups regarding the values of HW/BW and cardiac pathological scores (both *P* > 0.05).

### Neutralization of IL-38 regulated Th1 and Th17 cells differentiation in vivo

**Fig. 5:**
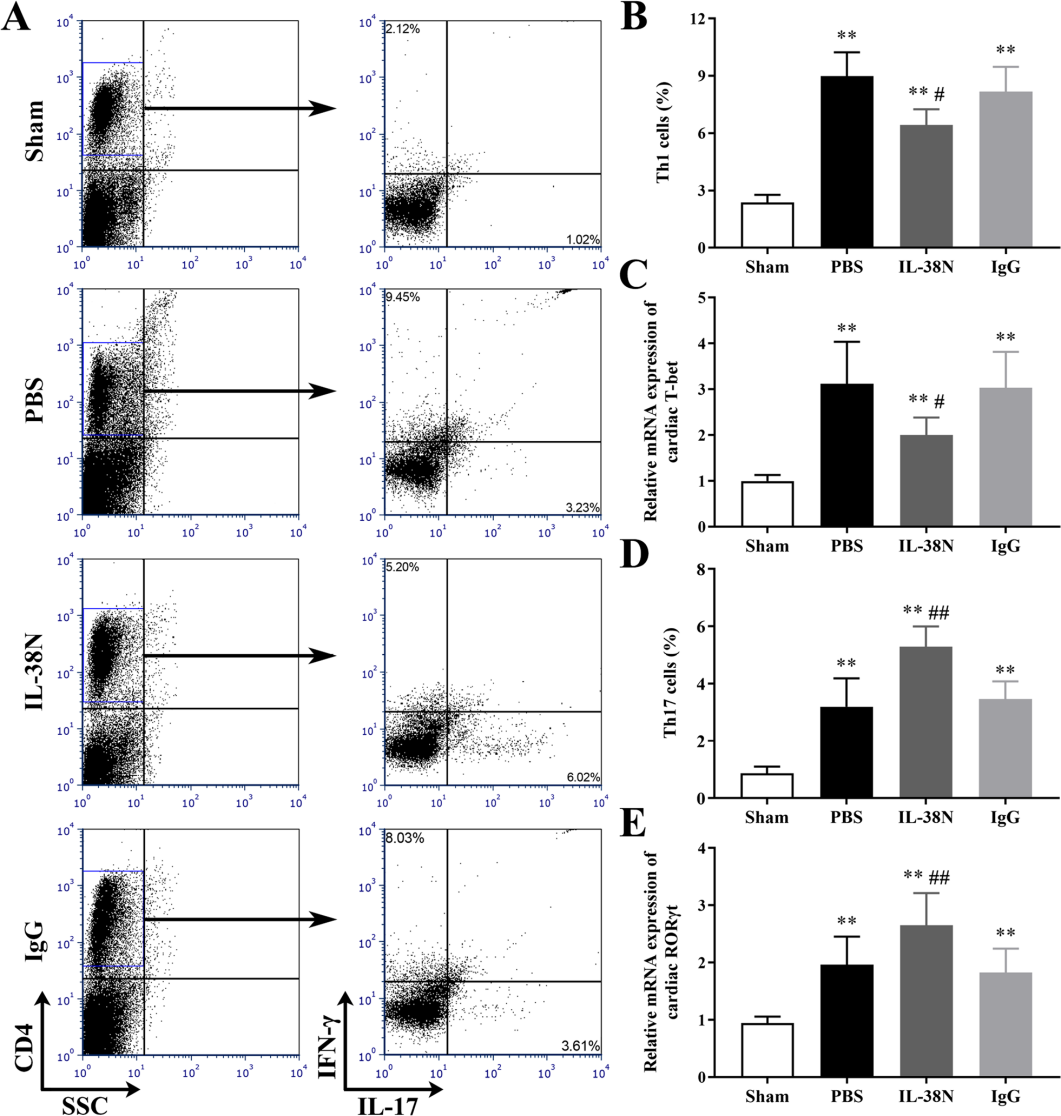
Neutralization of IL-38 regulated Th1 and Th17 cells differentiation in vivo. **A** Representative flow cytometry dot plots of splenic CD4^+^IFN-γ^+ ^Th1 cells and CD4^+^IL-17^+ ^Th17 cells in the Sham, PBS, IL-38N, and IgG groups. Numbers in upper left quadrants and lower right quadrants indicate the separate percentages of CD4^+ ^Th1 cells and CD4^+ ^Th17 cells. **B** Abundance of Th1 cells in different groups. **C** Cardiac mRNA expression levels of T-bet in different groups. **D** Abundance of Th17 cells in different groups. **E** Cardiac mRNA expression levels of RORγt in different groups. ^**^P < 0.01, compared to Sham group; ^#^*P* < 0.05, ^##^*P* < 0.01, compared to IgG group. Data are expressed as mean ± SD

Previous studies have suggested that Th cells differentiation and their production of cytokines, such as IFN-γ-producing Th1 cells and IL-17-producing Th17 cells, are involved in the pathogenesis of AVMC [7–9]. Moreover, there is growing evidence that IL-38 can mediate an effective immune response via the regulation of Th cells differentiation [12, 24]. Therefore, the percentages of splenic Th1 and Th17 cells, and the mRNA expression levels of master transcription factors (T-bet and RORγt) in myocardium were measured on day 7 post-infection. As shown in Fig. 5, the percentages of splenic Th1 and Th17 cells, and cardiac mRNA expression levels of T-bet and RORγt were markedly increased in the PBS, IL-38N, and IgG groups compared to those in the Sham group (all *P* < 0.01). Furthermore, compared to those in the IgG group, the percentages of Th1 cells in the IL-38N group were significantly diminished (Fig. 5A–B, *P* < 0.05), whereas the percentages of Th17 cells were significantly elevated (Fig. 5A and D, *P* < 0.01). Similarly, cardiac mRNA expression level of T-bet associated with Th1 cells was decreased in the IL-38N group compared to that in the IgG group, whereas RORγt associated with Th17 cells was increased (Fig. 5C and E, *P* < 0.05 or *P* < 0.01). No significant differences in the percentages of Th1/Th17 cells and the expression levels of transcription factors were observed between the PBS and IgG groups (all *P* > 0.05).

### Neutralization of IL-38 reduced IFN-γ production, increased IL-17, TNF-α, and IL-6 levels, and promoted CVB3 replication

**Fig. 6:**
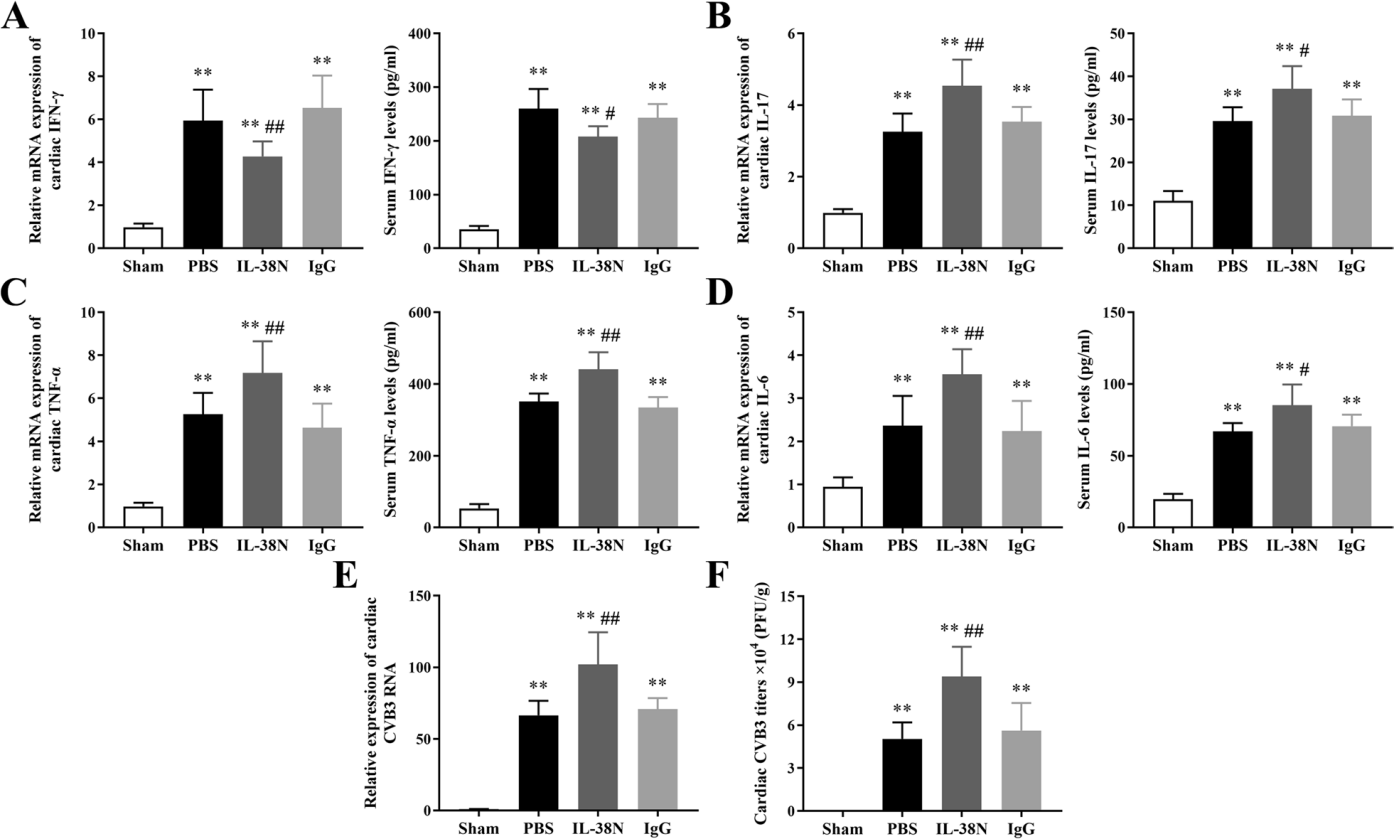
Neutralization of IL-38 reduced IFN-γ production, increased IL-17, TNF-α, and IL-6 levels, and promoted CVB3 replication. Cardiac mRNA expression levels of IFN-γ, IL-17, TNF-α, and IL-6 were detected by RT-qPCR, and serum protein levels of these cytokines were measured by ELISA in the Sham, PBS, IL-38N, and IgG groups. **A–D** IFN-γ, IL-17, TNF-α, and IL-6, respectively. **E** Levels of cardiac CVB3 RNA in different groups. **F** Levels of cardiac CVB3 titers in different groups. ^**^P < 0.01, compared to Sham group; ^#^*P* < 0.05, ^##^*P* < 0.01, compared to IgG group. Data are expressed as mean ± SD

Cardiac mRNA expression levels of Th1-related cytokine (IFN-γ) and Th17-related cytokines (IL-17, TNF-α, and IL-6) were detected by RT-qPCR. Additionally, serum protein levels of these cytokines were measured by ELISA. The mRNA and protein expression levels of IFN-γ, IL-17, TNF-α, and IL-6 were markedly higher in the PBS, IL-38N, and IgG groups than those in the Sham group (Fig. 6A–D, all *P* < 0.01). Compared to those in the IgG group, the mRNA and protein expression levels of IL-17, TNF-α, and IL-6 were significantly increased in the IL-38N group, in contrast to the levels of IFN-γ, which were decreased (Fig. 6A–D, *P* < 0.05 or *P* < 0.01). Moreover, cardiac CVB3 RNA levels and CVB3 titers in the IL-38N group were significantly elevated compared to those in the IgG group (Fig. 6E–F, all *P* < 0.01). No significant differences in the levels of these cytokines and CVB3 replication were detected between the PBS and IgG groups (all *P* > 0.05).

## Discussion

IL-38 has been considered as an IL-1 family antagonist for its high homology to IL-36Ra and IL-1Ra [10]. Most studies conclude that IL-38 exerts anti-inflammatory effects in several autoimmune diseases, including RA, AA, and SLE [13–15]. On the contrary, several other studies have shown that IL-38 can act as a pro-inflammatory factor, and it can modestly enhance candida-induced IL-22 and IL-17 expression in human PBMCs at a higher concentration [12, 25]. Truncated IL-38 and mature IL-38 have an anti-inflammatory effect, whereas full-length IL-38 needs to be cleaved in an apoptosis environment to exert its anti-inflammatory effect [26]. However, it has also been reported that knocking out IL-38 does not cause changes in pro-inflammatory cytokines and has no effect on imiquimod-induced psoriasis in mice [27]. Therefore, IL-38 can have pro-inflammatory or anti-inflammatory properties that are likely dependent on its concentration, form, and local environment. Its functional role in CVB3-induced AVMC, if any, is hence unclear. In this study, we provide, for the first time, evidence of significant elevation in cardiac mRNA expression and protein levels of IL-38, and serum IL-38 protein in AVMC. Cardiac mRNA expression of IL-36R, the specific receptor of IL-38, was also markedly up-regulated after CVB3 infection. Moreover, it is noteworthy that the increased IL-38 levels in myocardium and serum were negatively correlated with the severity of AVMC, suggesting that IL-38 may act as a protective agent against AVMC. Further research on IL-38 will help shed new light on the pathogenesis of AVMC.

Based on the observation that the levels of IL-38 and IL-36R were elevated in AVMC, and that the IL-38 levels showed a negative correlation with the severity of AVMC, we tentatively explored the potential role of IL-38 in a mouse model of AVMC with the use of Anti-IL-38 Abs in vivo. Our experimental results demonstrated that neutralization of IL-38 exacerbated the severity of AVMC, as verified by the lower survival rate, impaired cardiac function, continuous bodyweight loss, and higher values of HW/BW and cardiac pathological scores. Aberrant IL-38 expression has been reported in many organs and tissues, such as skin, spleen, placenta, tonsil, thymus, and salivary gland [11]. However, reports concerning the expression and function of IL-38 in inflammatory myocardial diseases remain scarce so far. Previous studies have shown that IL-38 can be a predictor of successful reperfusion and a diagnostic and prognostic marker in patients with myocardial infarction (MI) [28]. Recently, Wei and colleagues demonstrated that inflamed cardiomyocytes and infiltrated macrophages are the main cellular source of IL-38, and that IL-38 plays a protective effect in ventricular remodeling in mice with MI [29]. These findings, together with ours, suggest that IL-38 may be a novel therapeutic target for the treatment of inflammatory myocardial diseases.

In the early stage of CVB3 infection, direct viral invasion causes cardiomyocytes necrosis/apoptosis, which induces the infiltration of immune cells and cytokines production to regulate the host immune response. Th1 cells are known to play a crucial immunoregulatory role in both the innate and adaptive immune responses to viral infections, based on the production of IFN-γ, a broad-spectrum antiviral cytokine [7]. Recent studies indicate that Th17 cells, which secrete the characteristic cytokine IL-17, contribute to cardiac viral replication by inhibiting Th1 cells differentiation via IL-17 signaling and promote an autoimmune response in AVMC through the excessive release of pro-inflammatory cytokines [8, 30]. The role of IL-38 in regulating Th1 and Th17 cells responses has not been identified in AVMC. It has been reported that IL-38 exerts a protective role by preventing the activation and function of Th1 and Th17 cells in most inflammatory and autoimmune diseases [11]. However, Chu et al*.* found that IL-38 treatment attenuates the severity of SLE due to a reduction in Th17 numbers, with no influence on Th1 cells in mice [31]. Thus, it seems that the regulatory role of IL-38 on Th1 and Th17 cells is not entirely consistent across different disease models and requires further in-depth investigation. In this study, the infiltration of Th1 and Th17 cells, as well as the levels of related cytokines in myocardium and serum were increased in mice with AVMC. Additionally, the aggravating effect of neutralizing IL-38 on myocarditis was closely associated with Th1/Th17 cells imbalance and related cytokines production during the development of AVMC, which had not been reported before. T-bet and RORγt are the master transcription factors in directing differentiation of naïve CD4^+^ T cells to Th1 and Th17 cells, respectively [32]. The present study showed that IL-38 neutralization in vivo suppressed the expression of T-bet but promoted RORγt expression, suggesting that T-bet and RORγt may be the potential targets of IL-38 neutralization in inducing Th1/Th17 cells imbalance. In an in vitro study, Chai et al*.* demonstrated that Th17 cells differentiation and IL-17 production have no significant changes after the treatment with Anti-IL-38 Abs, while IL-38 recombination protein can significantly decrease percentages of Th17 cells through the suppression of p-STAT3 (another important transcription factor of Th17 cells) [33]. Since there is no expression of IL-38 in mouse CD4^+^ T cells, but mouse CD4^+^ T cells display surface expression of IL-36R (IL-38 specific receptor) [10, 11], making them capable of receiving the biological signal of IL-38 released by a large number of infiltrated immune cells (e.g., B cells, NK cells, macrophages, and DCs) during the acute phase of infection, this may be an important mechanism by which Anti-IL-38 Abs participate in the regulation of Th cells differentiation in vivo. More specifically, neutralization of IL-38 in the IL-38N group could significantly promote CVB3 replication, which was consistent with the previous observation that IL-38 contributed to resistance to viral infection [16–18], further highlighting an antiviral role for IL-38 in AVMC. We then conclude that IL-38 may play a role in regulating the balance between antiviral immunity and autoimmunity in CVB3-induced AVMC.

To our knowledge, this is the first report of IL-38 being involved in the pathogenesis of AVMC. However, the study has several limitations. First, all experiments were performed exclusively in male mice. Previous studies have shown that female mice are less susceptible to CVB3 infection and less prone to develop severe AVMC than male mice, mainly due to the protective effect of estradiol [34]. Therefore, to simplify the current study, we chose to focus on male mice. Whether Anti-IL-38 Abs have the same effect on AVMC in female mice requires further assessment. Second, it has been shown that IL-38 binds to IL-36R and exerts its effects by antagonizing the activation of intracellular signaling pathways such as JNK/AP1, p38 MAPK, ERK1/2, and NF-κB [14, 35], which have been widely confirmed to be involved in the pathogenesis of AVMC [2, 5, 6]. Studies on the downstream signaling pathway of IL-38 will help reveal its regulatory mechanisms in AVMC in more detail. Furthermore, recent studies have shown that IL-38 treatment can effectively reduce fibrosis in different animal models [29, 36]. The potential role of IL-38 in chronic myocarditis and dilated cardiomyopathy is still an interesting open issue.

## Conclusions

In summary, our preliminary data demonstrate that neutralization of IL-38 significantly exacerbated CVB3-induced AVMC in mice. Neutralization of IL-38 in vivo resulted in the imbalance of Th1/Th17 cells and increased CVB3 replication. Therefore, our investigation shows that IL-38 may play a myocardium-protective role in AVMC and suggests IL-38 as a potential therapeutic target.

## Supplementary Information

**Additional file 1: Figure S1.** Effects of different doses of Anti-IL-38 Abs on IL-38 expression in vivo. Male BALB/c mice were treated with three different doses of Anti-IL-38 Abs (25, 50, or 75 μg per mouse) by i.p. injection on day 0 and day 4 after CVB3 infection. Cardiac mRNA expression levels of IL-38 were detected by RT-qPCR (**A**), and its protein levels in serum were measured by ELISA (**B**) on day 7. Each dose group contained 5 surviving mice. ^**^P < 0.01, compared to mice without Anti-IL-38 Abs treatment; ^#^*P* < 0.05, ^##^*P* < 0.01, compared to mice treated with Anti-IL-38 Abs (25 μg per mouse per injection). Data are expressed as mean ± SD.

## Data Availability

The data used to support the findings of this study are included in this current manuscript and its additional files.

## Abbreviations

IL-38: Interleukin-38
AVMC: Acute viral myocarditis
CVB3: Coxsackievirus B3
IL-36R: Interleukin-36 receptor
Abs: Antibodies
Th: T helper
Ra: Receptor antagonist
PBMCs: Peripheral blood mononuclear cells
NK cells: Natural killer cells
DCs: Dendritic cells
RA: Rheumatoid arthritis
AA: Allergic asthma
SLE: Systemic lupus erythematosus
MI: Myocardial infarction
i.p.: Intraperitoneal
PFU: Plaque-forming unit
PBS: Phosphate-buffered saline
LVESD: Left ventricular end-systolic diameter
LVEDD: Left ventricular end-diastolic diameter
FS: Fractional shortening
EF: Ejection fraction
H&E: Hematoxylin and Eosin
IOD: Integrated optical density
RT-qPCR: Quantitative real-time PCR
HW/BW: Heart weight/bodyweight
