# Supplementary material for: Neutralization of interleukin-38 exacerbates coxsackievirus B3-induced acute myocarditis in mice
Source: Virol J. 2021 Nov 14;18:220. doi: 10.1186/s12985-021-01687-w (PMC8590870; doi:10.1186/s12985-021-01687-w)
Supplement: Supplementary file 1 [file 12985_2021_1687_MOESM1_ESM.docx]

**Figure S1. Effects of different doses of Anti-IL-38 Abs on IL-38 expression** ***in vivo*****.** Male BALB/c mice were treated with three different doses of Anti-IL-38 Abs (25, 50, or 75 μg per mouse) by i.p. injection on day 0 and day 4 after CVB3 infection. Cardiac mRNA expression levels of IL-38 were detected by RT-qPCR **(A)**, and its protein levels in serum were measured by ELISA **(B)** on day 7. Each dose group contained five surviving mice. ^**^*P*<0.01, compared to mice without Anti-IL-38 Abs treatment; ^**#**^*P*<0.05, ^**##**^*P*<0.01, compared to mice treated with Anti-IL-38 Abs (25 μg per mouse per injection). Data are expressed as the mean ± SD.
